# Preparation of a porous superhydrophobic foam from waste plastic and its application for oil spill cleanup

**DOI:** 10.1039/c9ra06848a

**Published:** 2019-11-19

**Authors:** Chuanming Yu, Wenyu Lin, Jine Jiang, Zhanxin Jing, Pengzhi Hong, Yong Li

**Affiliations:** Faculty of Chemistry and Environmental Science, Guangdong Ocean University Zhanjiang 524088 PR China yucmingdou@163.com yongli6808@126.com

## Abstract

In order to cope with the increasing oil spill accidents and the intentional discharge of oily wastewater, a new oil-adsorbing material with superhydrophobicity and reusability is needed. In this paper, waste plastic was used to fabricate an alveolate polystyrene (PS) foam to reduce secondary pollution. The PS foam was synthesized from a high internal phase Pickering emulsion (HIPPE) technique in a one-step process. The emulsion was stabilized by a co-Pickering system of Span 80 surfactant and SiO_2_ particles. To explain the super stability of the HIPPE, a novel model of the water-in-oil droplet was promoted. The obtained SiO_2_@PS foam exhibited a multi-order-porous structure, and displayed superhydrophobicity and superoleophilicity. It can selectively remove various oily contaminants from water with a high adsorption capacity of 20.4–58.1 g g^−1^ at a fast rate. The oil-adsorbed material can be reused by simple centrifugation, and no more than a 1% decline was obtained in the oil adsorption after 10 cycles. Therefore, the SiO_2_@PS foam has a great potential application in oily water treatment.

## Abstract 2

In this paper, waste polystyrene (PS) plastic was used to fabricate alveolate PS foam *via* a high internal phase Pickering emulsion (HIPPE) which was stabilized by Span 80 and silica particle as a co-Pickering emulsifier in one-step progress.

## Graphical abstract

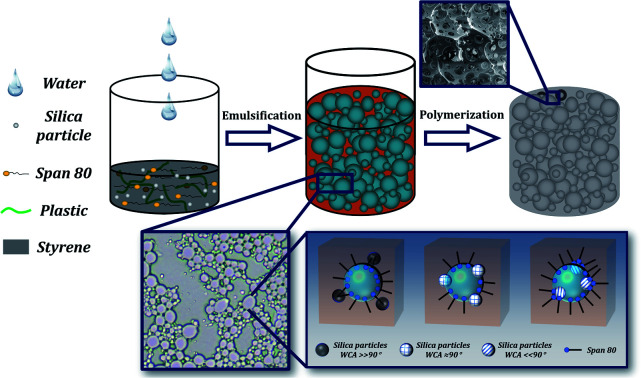

## 1. Introduction

Over the past few decades, petroleum energy has been widely used since the development of humanity stepped into the industrial age. During the exploitation or transportation of oil, immediate oil spill occurs frequently, causing serious damage to water resources. This not only brings huge economic losses, but also destroys the ecological environment and risks human health. Oil pollution in water sources has become one of the major environmental problems that need to be solved globally.^1^

At present, the common methods employed to deal with oil spills include physical adsorption,^2^ chemical treatment^3^*in situ* combustion^4^ and biotechnology.^5^ Physical adsorption is considered as an economical method to collect the spilled oil because it is simple to operate and avoids subsequent contamination of the environment. When processing with physical adsorption, it is important to select a suitable adsorbent material. A porous polymeric composite is deemed asan effective material because it is easy to prepare and recycle, and has a satisfactory adsorption capacity.^6–8^ To date, numerous polymers have been employed as the oil adsorbents, such as foams,^9,10^ resins,^11–14^ sponges,^15^ and aerogels.^16,17^ In order to allow the material to selectively adsorb the oil in the oil–water mixture, the ideal adsorbent material should have an excellent hydrophobic and oleophilic surface. For instance, Wang *et al.*^18^ fabricated superhydrophobic–superoleophilic polydopamine (PDA)- and dodecanethiol (DDT)-coated melamine (MF) sponge (PDA/DDT@MF) through a facile self-polymerization. The resultant PDA/DDT@MF could selectively adsorb various oils or organic solvents with the adsorption capacity being up to 98.6 times over its own weight. Zhang *et al.*^19–21^ prepared a polystyrene (PS) foam by a template polymerization from a high internal phase emulsion (HIPE), in which Fe_3_O_4_ nanoparticles or carbonyl iron powders were added to stabilize the emulsion and bring magnetic responsiveness to the obtained materials. The magnetic foam exhibited a high oil/water selectivity with oil adsorption of 57 g g^−1^. Pan *et al.*^22^ obtained a TiO_2_ nanoparticle/polyurethane (TPU) sponge coated by tetradecylamine (TDA) amidated graphene oxide (GO-TDA), which provided a novel strategy for oil/water separation. TPU-GO-TDA displayed excellent superhydrophobicity and superoleophilicity with a high oil sorption capacity of 62.4 g g^−1^. However, the aforementioned methods involved the use of expensive chemicals in the preparation of the materials, which may also bring secondary pollution to the ecological environment, thus making them less competent for practical industrial oil–water separation.

Another issue that deserves attention is the large amount of plastic that is currently polluting the environment we are living in, such as land, air, waterways, and oceans. As of 2018, approximately 380 million tons of plastic are being produced worldwide each year. From the 1950s to 2018, an estimated 6.3 billion tons of plastic has been produced worldwide, of which about 12% has been burned and only 9% has been recycled.^23^ The discarded plastics not only endanger human survival, but also pose a serious threat to the survival of animals, birds, fishes, and wildlife. Since the development of the plastics industry, there has not been an effective method for rapidly degrading these plastic wastes. The chemical structure of most plastics renders them resistant to many natural processes of degradation, and as a result, they are slow to degrade. Moreover, low price and durability resulted in a high level of production of plastics. Together, these two features have brought about a high prominence of plastic pollution in the environment.

Herein, we first propose the secondary use of waste polystyrene plastic for the preparation of an oil-adsorbing polymer foam. The foam was manufactured *via* a one-pot high internal phase Pickering emulsion. This method was accessible and convenientto obtain the foam without freezing, vacuum drying, or some other complex procedures. Additionally, silica and Span 80 were employed as a co-Pickering emulsion system to stabilize the emulsions, in which the waste packaging foam was added as a crosslinker due to its low surface energy. Low surface energy is beneficial for the discarded PS plastic to be dissolved in a styrene monomer and construct a hydrophobic skeleton of the target material. Hence, the obtained foam demonstrated superhydrophobic and oleophilic performance and could be used to separate the oil spills and heavy oil from water. The water amount control experiments showed that the amount of water in the preparation of the material significantly affected the oil adsorption capacity of the foam. Moreover, the foam could be reused for at least 10 cycles of oil adsorption-recovery, thus achieving a stable performance and proving that the material can be used as an efficient adsorbent for oil spill cleanup.

## 2. Experimental

### 2.1 Chemicals and materials

The discarded PS plastic was collected from packages, and the dust was removed by washing with deionized water. Then, the discarded PS plastic was soaked in ethanol for 24 h to remove organic pollutants. Finally, the experimental plastic was obtained by drying at 30 °C to achieve constant weight. Styrene, tetraethylorthosilicate (TEOS) and hexadecyltrimethoxysilane (HTOS) were purchased from Shanghai Aladdin-e Reagent Co. Ltd. Ammonia solution (25–28 wt%) and isopropyl alcohol were purchased from Guangdong Guanghua Sci-Tech Co., Ltd. Other reagents used in the paper were purchased from Shanghai Lingfeng Chemical Reagent Co. Ltd. All the reagents were directly used without any further purification.

### 2.2 Preparation of silica particles

To a stirred solution of 100 mL isopropanol, 8 mL tetraethylorthosilicate (TEOS), 1 mL hexadecyltrimethoxysilane (HTOS) and 20 mL 28% ammonia solution were added, in sequence. After 6 hours of reaction, the mixture was centrifuged, and the supernatant was decanted. The remaining solid was washed with deionized water and ethanol 3 times, respectively, then transferred to an oven, dried at 100 °C for 6 h, and ground to obtain the superhydrophobic silica powder. Hydrophilic silica particles were prepared in the same manner as above without HTOS.

### 2.3 Preparation and polymerization of HIPPEs

Based on our previous study,^24^ we improved the preparation method of the poly-HIPPE. First, the viscous HIPPE was obtained, in which the organic phase was prepared as follow: 0.25 g shredded packaging polystyrene foam was dissolved by using 0.50 mL styrene in a 20 mL Cillin bottle; then, 0.02 g of a free radical emulsifier initiator AIBN and a co-Pickering emulsion stabilizer of 0.02 g SiO_2_ nanoparticles (20–30 nm) and 0.03 g Span 80 were added with ultrasonication for 10 min. To the uniform suspension, deionized water was added stepwise. The oil–water mixture was then vigorously shaken with hand to obtain a sticky and homogeneous HIPPE. It should be noted that it is necessary to ensure that the system is a viscous emulsion before each addition of water. Further, the generated final HIPPE was put into an oven, and the organic phase was polymerized at 75 °C for 8 h. The obtained material was removed from the bottle carefully, and then dried at 45 °C to constant weight. Following this, the residual organic monomers and Span 80 surfactant in the polymer were removed by Soxhlet extraction with ethanol for 8 h. Finally, the resulting foam was dried at 75 °C for 2 h to achieve constant weight.

### 2.4 Characterization of foams

The foam was cut into small pieces and a MIRA3 field-emission scanning electron microscope (SEM, TESCAN, Czech) with an accelerating voltage of 5.0 kV was operated to investigate the microstructure of the foam after being sputter-coated with gold powder. The pore size distribution of the foam was measured by mercury porosimetry (Autopore IV, Micromeritics, USA). A commercial XG-CAM7.1 optical system was used to measure the contact angle (CA) of the foam through the sessile drop method. The droplet volume in the experiment was about 6–8 μL, and at least three different points on the same sample surface were tested for contact angle measurements (XG-CAMB3) to receive an average value.

### 2.5 Porosity measurement

The porosity (average open porosity) and density of foams were determined by a liquid displacement method with chloroform as the displacement liquid because it can easily penetrate the pores of foams and did not induce shrinkage or swelling as a nonsolvent of the polymers. The density of chloroform (*ρ*_c_) at 25 °C is 1.483 g cm^−3^. All the samples were tested in triplicate. First, the foam samples were cut into round pieces with diameter of ∼20 mm, and volumes (*V*) of the samples were measured *via* a draining method because the foam shows excellent hydrophobicity; then, the initial weight (*W*_0_) of the foam was measured. Second, the foam was immersed in chloroform for 1 h to facilitate chloroform transport into the pores of the material. The final weight (*W*_1_) of the foam was noted and porosity was calculated according to the equation below:^25^
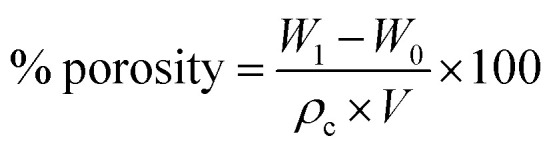


The density of the foam, *ρ*, was expressed as below:^26^
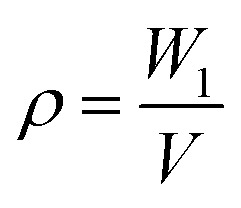


### 2.6 Oil adsorption

Oil adsorption was performed by dipping porous PS foam composite samples into the oil (including chloroform, acetone, hexane, dichloromethane, acetic ether, methanol, ethanol, toluene, peanut oil, diesel, pump oil, and crude oil). The oil-adsorbed foam was determined by a gravimetric method. In a typical adsorption process, approximately 0.1 g of the dried foam sample in cylindrical shape was immersed in oil at room temperature for 5 min. Following this, the sample was taken out from the oil, and then weighed immediately. The oil adsorption was calculated according to the following formula:^19^*K* (g g^−1^) = (*m*_1_ − *m*_0_)/*m*_0_where *K* represents the oil adsorption capacity of the foam, *m*_1_ is the weight of the foam at saturation adsorption, and *m*_0_ is the initial weight of the foam. For each sample, the oil adsorption was repeated three times, and an average value was calculated as the final oil adsorption.

### 2.7 Oil retention and recovery

The fully oil-adsorbed PS foam composite was weighed as *m*_1_. Then, the oil was removed by simple centrifugation at a speed of 3000 rpm for 10 min, and the residual foam was weighed as *m*_2_. The oil retention was calculated by using the following formula:^24^*R* (%) = (*m*_1_ − *m*_2_)/*m*_2_ × 100

The residual foam composite was directly used to retest the saturated oil adsorption for reutilization.

## 3. Results and discussion

### 3.1 The preparation of the emulsion

**Fig. 1:**
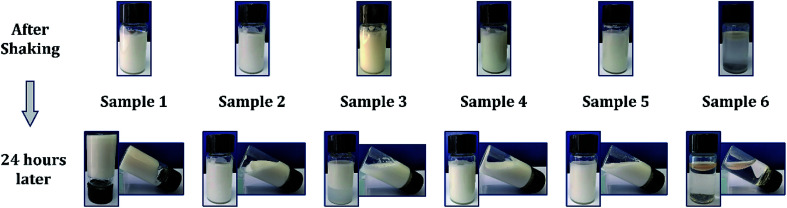
The emulsion prepared based on Table 1.

In the preparation of HIPPEs, there are two functions for styrene: as the solvent for waste plastics and as the monomer for polymerization to construct the skeleton of new materials. A series of emulsions were prepared by controlling the amount of the stabilizer, according to Table 1. As shown in Fig. 1, sample 3 was prepared with Span 80 as an emulsifier singlehandedly and turned into two layers after 24 hours, showing that using only Span 80, it is difficult to maintain a HIPE. The reason may be that the water content in the oil–water system was high, the volume ratio was up to 94.1%, and the amount of emulsifier Span 80 was very small, due to which it was difficult to maintain the stability of the system. Similarly, for sample 6, it was observed that an individual silica particle can only emulsify a small portion of the oil–water mixture, but the emulsion will quickly return to the oil–water biphasic system, while the silica disperses in the upper oil phase. Exhilaratingly, the silica particle and Span 80 co-stabilized emulsion showed excellent stability, and the stability could be adjusted by changing the amount of Span 80 or silica. Compared to sample 3, the stability of sample 2 was significantly improved after adding 0.01 g of SiO_2_. Furthermore, by increasing the amount of silica to 0.03 g, it was found that the emulsion still showed satisfactory viscosity after being left for 24 hours. The results indicate that with the assistance of Span 80, the stability of the emulsion can be improved by increasing the content of silica particles. By studying the appearance of the emulsions of samples 1, 4 and 6, it was also concluded that more Span 80 was more favorable for stabilizing the HIPPEs in the presence of silica. Therefore, a very small amount of silica particles combined with Span 80 can easily achieve a steady emulsification of the oil–water mixture. This synergistic stabilizing effect replaces part of the organic emulsifiers with inorganic particles, reduces the content of organic components and their environmental hazards, and may also facilitate the acquisition of some complex emulsions.

**Table 1:** Composition of the emulsions

Sample	H_2_O (mL)	Span 80 (g)	Hydrophobic SiO_2_ (g)	Visual quality	
				After shaking	24 hours later
1	8	0.3	0.03	Sticky	Sticky
2	8	0.3	0.01	Sticky	Soft, fluid
3	8	0.3	0	Fluid	Two zones (w, e)
4	8	0.1	0.03	Sticky	Soft, fluid
5	8	0.1	0.01	Sticky	Soft, fluid
6	8	0	0.03	Two zones (w, o)	Two zones (w, o)

**Fig. 2:**
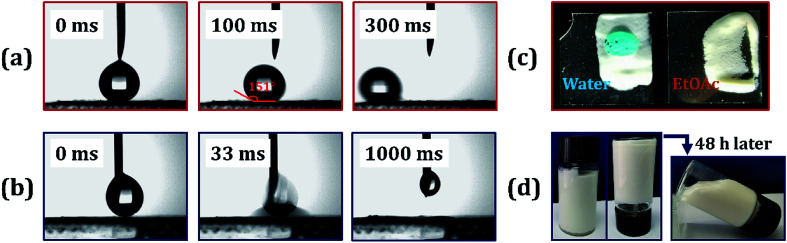
(a) WCA of hydrophobic silica of 151°; (b) WCA of hydrophilic silica approaching 0°; (c) dyeing experiment of emulsion. For clearer observation, water was dyed with MB and oil (ethyl acetate) was dyed with Sudan II; (d) emulsion prepared with hydrophilic silica and Span 80 as a co-Pickering emulsifier.

According to Finkle *et al.*,^27^ for a Pickering emulsion, the liquid that does not easily wet the particles tends to be a dispersed phase. Therefore, hydrophobic particles can be used to stabilize the water-in-oil (W/O) system, and hydrophilic particles are mainly used to form an oil-in-water (O/W) emulsion. Here, the silica particles we used exhibited excellent hydrophobicity, as shown in Fig. 2(a), and the water contact angle (WCA) was about 151°. To investigate the type of the prepared emulsion, a dyeing experiment was performed. As shown in Fig. 2(c), a drop of methylene blue (MB)-stained water was placed on the surface of the emulsion, and it was observed that the water droplets did not disperse. On the contrary, Sudan II-dyed ethyl acetate was dispersed immediately. These results indicate that the emulsion prepared in this experiment is a W/O emulsion. Furthermore, we performed an experiment by using hydrophilic silica instead of hydrophobic silica, as shown in Fig. 2(b), and found that a stable high internal phase emulsion could also be obtained, as shown in Fig. 2(d). The dyeing experiment confirmed that the emulsion was also a W/O emulsion, which was inconsistent with Finkle *et al.*'s conclusion. This may be related to the stability mechanism of the emulsion *via* the Pickering emulsifier.

### 3.2 The stability mechanism of emulsion

**Fig. 3:**
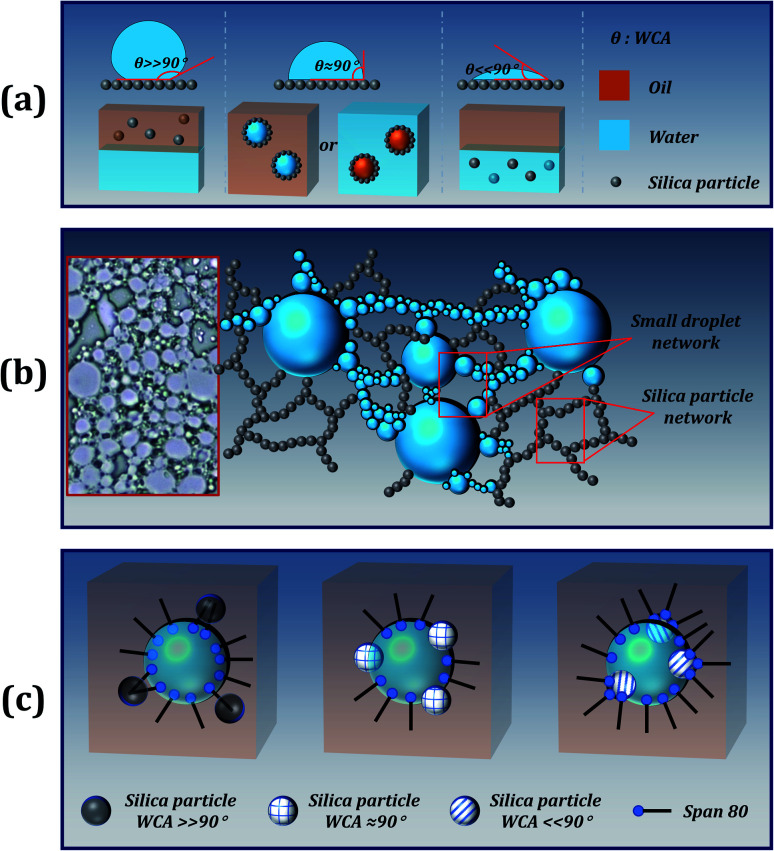
(a) Dispersion of different wettability silica particles in an oil–water system; (b) mechanism of stabilizing an emulsion by a hydrophobic silica particle; the inset illustration is the optical image of sample 1; (c) different models of W/O emulsions co-stabilized by Span 80 and silica particles with different wettability.

For particle-stabilized Pickering emulsions, the wettability of the particles is a key factor in effectively stabilizing the emulsion. In general, when solid particles were solely used (without Span 80 surfactant) as stabilizers, particles with WCA close to 90° endow the best stability to the emulsion. Since the particles are wettable to both water and oil, they can be stably dispersed at the interface of oil and water, as shown in Fig. 3(a). Moreover, stripping such particles from the interface requires higher desorption energy, making this stability irreversible. Therefore, Pickering particle-stabilized emulsions tend to have better stability than small-molecule stabilized emulsions. However, in order to prepare a stable high internal phase Pickering emulsion (HIPPE), a large number of solid particles are required, and the size and wettability of the particles also need to be modified. Fortunately, the addition of a small molecule emulsifier (Span 80) can solve the problem.

In the case of the addition of a small amount of Span 80 surfactant, competitive adsorptions of the surfactant molecules exist at the particle surface and at the W/O droplet interface. The presence of surfactant molecules facilitates the formation of small droplets during the emulsification process. At the same time, the interfacial tension was still high, and relatively large droplets were formed in the emulsion. As shown in the optical microscopy images of Fig. 3(b), fine droplets can flocculate and form bridges between the adjacent large droplets. The proposed explanation is in good agreement with the reported results.^28,29^ On this basis, the addition of silica particles creates a steric protective shield for emulsion droplets.^30^ Moreover, due to the excellent hydrophobicity and lipophilicity, the silica particles can be uniformly dispersed in the continuous phase to form a 3D network structure, which offers good stability to the emulsion, as presented in Fig. 3(b).^31^ Unfortunately, this mechanism cannot explain why superhydrophilic particles can enhance the stability of the emulsion. We speculate that there may be some interaction between the Span 80 surfactant and the particle, allowing the surfactant to be embedded on the surface of the silica particle to form an amphiphilic structure, similar to the Janus particles, as shown in Fig. 3(c). Furthermore, the hydrophilic silica particle with the WCA approaching 0° combines with the hydrophilic part of the Span 80 surfactant and creates a Janus particle. One side of the Janus particle is hydrophilic and the other side is hydrophobic, which allows the particles to be easily dispersed at the oil–water interface. Moreover, Span 80 with a low HLB value of 4.3 is a lipophilic emulsifier, which promotes the formation of a stable water-in-oil emulsion.

### 3.3 The morphology of foam

**Fig. 4:**
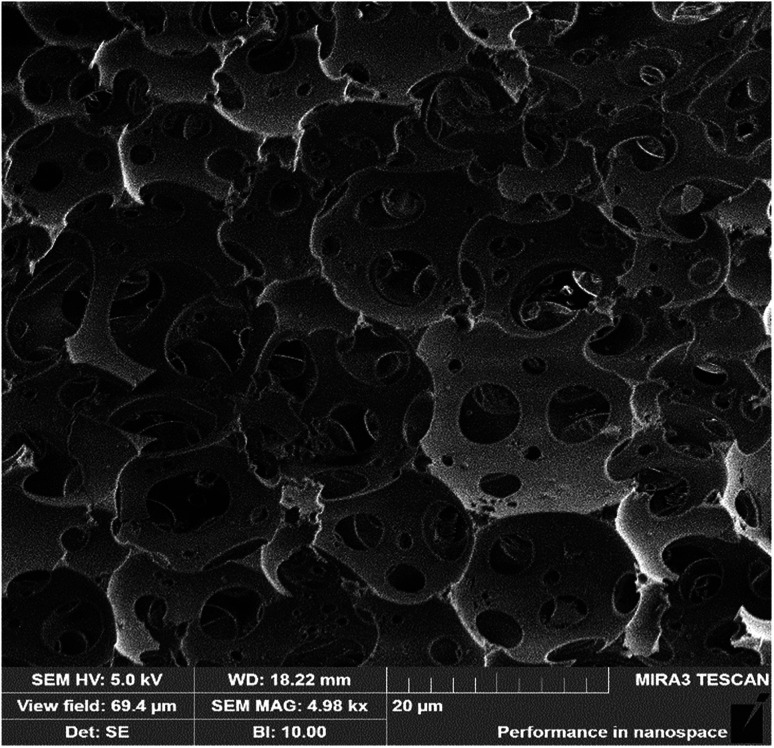
SEM image of sample 12.

**Fig. 5:**
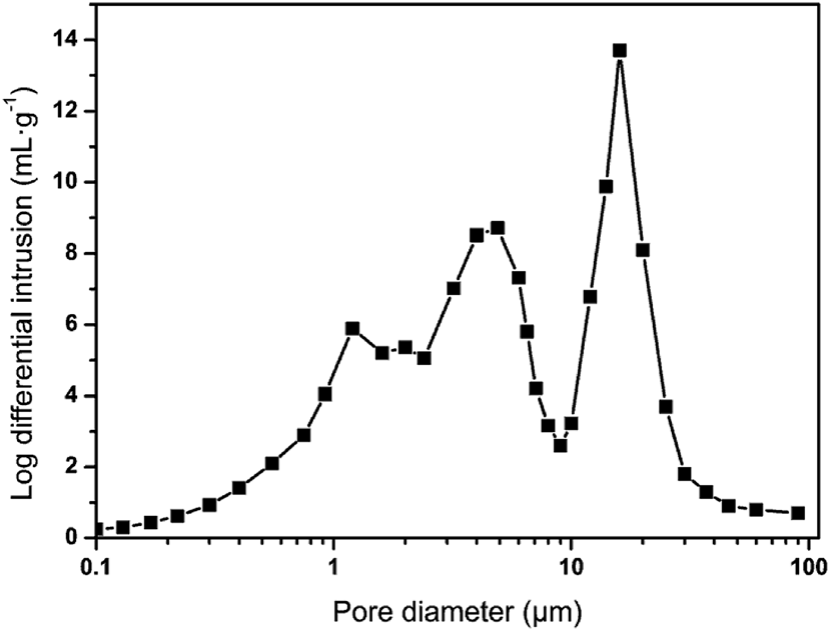
Pore size distribution of sample 12.

With the collective support of Span 80 surfactant and silica, above 88% (v/v) of water was equably dispersed in the HIPPE to construct a water-in-oil system. The styrene phase underwent free radical polymerization under the action of the AIBN initiator to form a skeleton of the material. When the moisture inside the material evaporated at 45 °C, the cellular structure was finally generated, as shown in Fig. 4. A high-magnification SEM image shows that the foam consists of multi-level pore characteristics. To investigate the pore size distribution of sample 12, a mercury intrusion instrument was employed, and the result is displayed in Fig. 5. Three evident peaks could be observed, and the peaks at around 5 μm and 16 μm were ascribed to the first-order pores, whereas the peak in the range from 1 μm to 2 μm was mainly attributed to the windows on the skeleton of the foam. The pore size distribution result was well-consistent with the SEM image. It was believed that the bigger pores were formed from the droplets and the window-holes were generated due to the shrinkage in the cavity during polymerization^32,33^ and fracture in desiccation.^34^ After a large number of trials, increasing the amount of Span 80 surfactant was found to bring about a significant increase in the quantity of window-holes. This phenomenon depends on the thickness of the oil film in the micelle. Williams believes that more surfactants facilitate the formation of smaller and more water-in-oil micelles, which increase the specific surface area of the oil film while reducing the thickness of the oil film. During the polymerization and drying process, the oil film will tend to crack at the weakest point to form a window aperture. Similarly, as the content of dispersed-phase water increases, the material tends to form more window-holes. Moreover, the increase in water volume is also beneficial to increase the porosity of the foam. It is foreseeable that higher porosity and more window-holes are beneficial to the oil adsorption properties of the material. With the increase in water volume from 3 mL to 18 mL, the density of the materials significantly decreased from 0.182 g cm^−3^ to 0.036 g cm^−3^ while the open porosity increased from 83.6% to 96.9%, as displayed in Table 2. Due to the possible presence of closed cells inside the material, the experimental value is always lower than the theoretical value. As a result, the density and porosity of the foam can be adjusted by varying the amount of emulsifier and dispersed phase.

**Table 2:** Data of foams^a^ with different water contents

Sample	H_2_O/mL	Density/g cm^−3^		Porosity/%	
		Theor.^b^	Exptl	Theor.^c^	Exptl
7	3	0.206	0.182	88.2	83.6
8	6	0.109	0.093	93.8	89.4
9	9	0.074	0.068	95.7	92.6
10	12	0.056	0.051	96.8	94.1
11	15	0.045	0.042	97.4	95.8
12	18	0.038	0.036	97.8	96.9

a The foams were fabricated *via* the polymerization of HIPPEs, which were stabilized by Span 80 and hydrophobic SiO_2_ particles.

b Theoretical density: calculated based on the total volume of water and oil, and the total mass of oil and solid particles.

c Theoretical porosity: calculated based on the volume of used water and oil.

### 3.4 The hydrophobicity and lipophilicity of foams

**Fig. 6:**
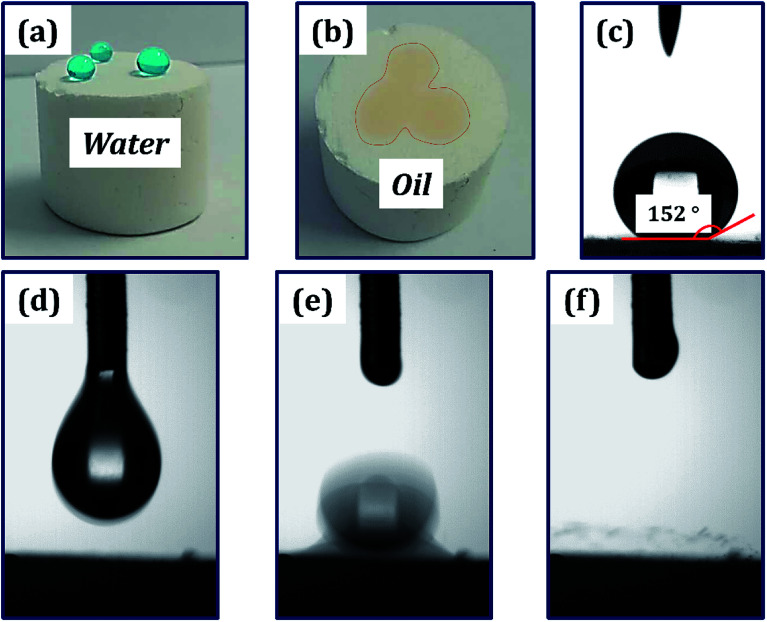
(a) Methylene blue-dyed water curled into a sphere on the surface of the foam. (b) Sudan II-dyed acetic ether adsorbed into the polyporous foam. (c) WCA of the HIPPEs foam. droplet size, ∼8 μL. (d–f) Different time photography of acetic ether droplets adsorbed into the polymer material: (d) 0 ms, (e) 32 ms and (f) 64 ms. Droplet size, ∼6 μL.

The polymer foam exhibited excellent hydrophobicity with a WCA equivalent to 152°, as shown in Fig. 6(c). Fig. 6(a) shows that the water droplets were absolutely standing on the surface of the foam like a sphere, and the droplets could easily slip off when subjected to slight external force or imbalance with an angle more than 7°. Moreover, the foam displays superlipophilicity with a contact angle between ethyl acetate and material surface approaching 0°. When a drop of Sudan II dyed ethyl acetate was placed on the monolithic surface, the oil was fleetly adsorbed, as shown in Fig. 6(b). The droplet began to touch the poly-HIPPEs at 0 ms. Once they were in contact, the oil was adsorbed frequently by the material in about 64 ms, indicating excellent lipophilicity of the obtained foam. Based on such excellent lipophilicity and hydrophobicity, the foam was expected to selectively adsorb oil from oily water.

### 3.5 The application in oil–water separation

**Fig. 7:**
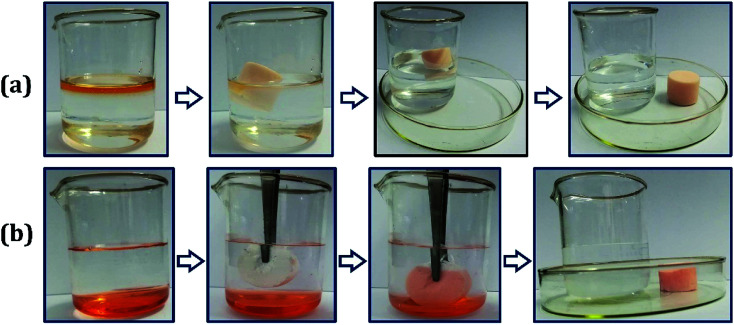
Oil–water separation images by the poly-HIPPE toward (a) acetic ether; (b) chloroform. Both of the organic liquids were dyed with Sudan II for a clear observation.

As shown in Fig. 7, the oil removal experiment was operated to investigate the application value of the oil-adsorbing poly-HIPPEs in the field of oil–water separation. Sudan II-dyed ethyl acetate and dichloromethane were used as the model oil pollutants. About 10 mL of ethyl acetate was placed on the surface of the water layer, and a regular cut foam was thrown in. It was observed that the poly-HIPPEs expeditiously adsorbed the oil, and the water turned clear, as shown in Fig. 7(a). Then, approximately 10 mL of dyed dichloromethane was dropped into the water and it quickly sank to the bottom. The dry foam was added into the mixture. It was observed that the foam floated on the water surface due to its superhydrophobicity and low density. When it was immersed below the water *via* tweezers, the foam palpably repelled water. Once it contacted with the bottom oil, the foam instantly adsorbed the oil and emitted the internal air. In a few seconds, the oil was completely adsorbed and clean water was finally obtained, as shown in Fig. 7(b). The oil removal experiment further indicates that the obtained foam possesses excellent lipophilicity and hydrophobicity, which can be applicated in sudden oil spill accidents and purification of oily wastewater.

### 3.6 Oil adsorbency and adsorption kinetics

**Fig. 8:**
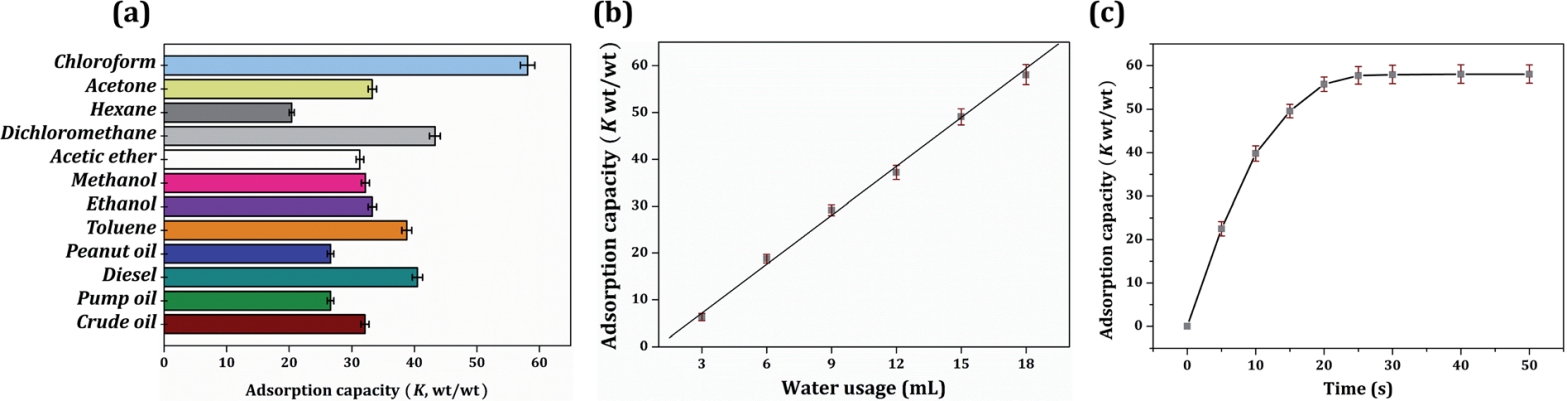
(a) Different oil adsorption capacity of sample 12; (b) chloroform adsorption capacity of samples 7–12; (c) adsorption kinetics of sample 12.

Saturated oil adsorption capacity is the core feature of the oil–water separation application. As shown in Fig. 8(a), the saturated oil adsorption capacities of the foam composite towards chloroform, acetone, hexane, dichloromethane, acetic ether, methanol, ethanol, toluene, peanut oil, diesel, pump oil, and crude oil were 58.1, 33.3, 20.4, 43.3, 31.3, 32.2, 33.3, 38.8, 26.6, 40.5, 26.6 and 32.1 g g^−1^, respectively. The oil adsorption capacity was higher than that reported for similar-functioning foams, sponges and resins.^21,35,36^ A series of foams were obtained by modifying the content of dispersed water (water volume ranging from 6 mL to 18 mL), and they expressed a significantly regular distinction in oil adsorption capacity. The increase in water content was clearly found to improve the oil adsorption capacity. This result is related to the composition of the HIPPEs, in which the dispersed water was evaporated to create the voids, and the voids were arranged to hold the organic liquid. Hence, more water usage creates larger pores and brings a greater oil adsorption capacity. In addition, there was a linear relationship between oil adsorption capacity and water usage, as shown in Fig. 8(b). Taking the adsorption capacity of chloroform as an example, the adsorption capacity (*K*) and the water consumption (*w*) were in accordance with the equation *K* = 3.484*w* − 3.309. The linear relationship was automatically fitted by the software Origin 7.5; the *R*-square was greater than 99.99% and the *P* value was less than 0.01%, indicating a good linear correlation.

It had been reported that the driving force for the oil adsorption rate was mainly owing to the capillary force and van der Waals force between the polymer molecule and oil droplet. Therefore, the foam is always designed to be hydrophobic for a better affinity with oil. Besides, according to Flory's swelling theory, specific surface area, void volume and void structure type (open pore or closed pore) also affect the adsorption behavior of the material.^37^Fig. 4(b) displays the chloroform adsorption kinetic curve. The poly-HIPPE sample 12 adsorbed chloroform very quickly in the first 10 seconds; then, the adsorption rate gradually slowed down toward steady state. In just about 30 seconds, the material can achieve oil saturation. The adsorption rate was much faster than that of similar oil-adsorbing materials reported, such as resins, sponges, and foams, prepared by the impregnation method.^38–40^ In this study, the fast adsorption speed was quite a delightful feature. It was mainly attributed to the widely distributed open-pore structure and the superlipophilicity of the entire material.^41^ However, it should be noted that excess water will lead to the demulsification of HIPPEs. Therefore, 18 mL (36 times volume of monomers) of water was the most suitable amount for this study.

### 3.7 Oil retention and regeneration

**Fig. 9:**
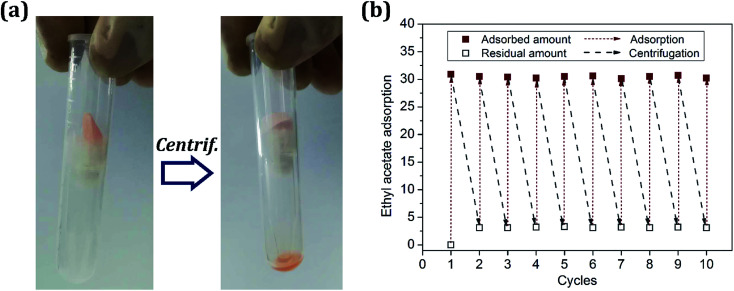
(a) Regeneration of the poly-HIPPE foam; (b) ethyl acetate adsorption capacities in different cycles.

High oil retention is a necessary property as an oil-adsorption material for accident oil treatment. Fig. 9(a) presents the regeneration of the foam *via* simple centrifugation at a speed of 2500 rpm for 5 min. In the adsorption/desorption cycles, acetic ether was chosen as the oil model to test the oil retention due to its low toxicity. The resulting poly-HIPPE samples 7–12 displayed oil retention values all above 90%. This means that most of the adsorbed oil could remain in the material. The high oil retention rate will facilitate the commercial application of materials. After regeneration, the residual foam was directly used for the next adsorption process. It was found that the adsorption capacity remains above 90% of the initial value. About 1% decline in the oil adsorption capacity of sample 12 was detected after 10 cycles, as presented in Fig. 9(b). Moreover, after 36 cycles, the oil adsorption reduced by 10%, which may have occurred because the repeated oil adsorption and centrifugation experiments caused the internal structure of the material to collapse and thus, the oil could not be locked inside. Besides, after each centrifugation, a certain amount of residual oil was always surviving in the foam. It is assumed that the residue was firmly adsorbed in the micropores due to the famous oleophilic nature of the material. Consequently, the poly-HIPPE displayed excellent reusability, suggesting that it is expected to remove oily contaminants from water.

## 4. Conclusions

In summary, the alveolate polystyrene foam was successfully fabricated from waste plastics *via* a high internal phase Pickering emulsion (HIPPE), which was stabilized by Span 80 and silica particles as a co-Pickering emulsifier in a one-step process. The emulsion preparation experiment indicated that a small amount of Span 80 and silica particles is much more beneficial for improving the stability of emulsion than a single stabilizer. The usage of waste plastic for oily water treatment is in accordance with green chemistry principles. A novel model of a water-in-oil droplet stabilized by Span 80 and SiO_2_ with different wettability was promoted to explain the super stability mechanism of the HIPPE. The obtained foam showed a multi-order-porous structure at the micro-nanometer level and displayed superhydrophobicity with the WCA approaching 152° and a superoleophilicity, which made the foam achieve the saturated adsorption in only 30 s. The optimized SiO_2_@PS foam exhibited adsorption capacity up to 20.4–58.1 g g^−1^ towards several oil models. The foam composite can selectively adsorb oil from oily water, and the adsorption capacity remains above 90% after 10 cycles, suggesting its outstanding reliability and reusability. Therefore, the SiO_2_@PS foam derived from waste plastics has great potential commercial applications in the field of oil spill cleanup.

## Conflicts of interest

There are no conflicts to declare.

## Supplementary Material
